# CPX-351 and allogeneic stem cell transplant for a therapy-related acute myeloid leukemia that developed after treatment of acute promyelocytic leukemia: a case report and review of the literature

**DOI:** 10.3389/fonc.2023.1291457

**Published:** 2024-01-25

**Authors:** Alessandra Sperotto, Maria Teresa Lupo Stanghellini, Jacopo Peccatori, Roberta De Marchi, Simona Piemontese, Giulia Ciotti, Marco Basso, Elisabetta Pierdomenico, Paolo Fiore, Fabio Ciceri, Michele Gottardi

**Affiliations:** ^1^ Onco Hematology, Department of Oncology, Veneto Institute of Oncology, Istituto Oncologico Veneto-Istituto di Ricerca e Cura a Carattere Scientifico (IOV-IRCCS), Castelfranco Veneto, Italy; ^2^ Hematology and Hematopoietic Stem Cell Transplantation Unit, Istituto di Ricerca e Cura a Carattere Scientifico (IRCCS) San Raffaele Scientific Institute, Milano, Italy

**Keywords:** acute promyelocytic leukemia, therapy-related myeloid neoplasm, allogeneic hematopoietic stem cell transplantation, CPX-351, acute myeloid leukemia

## Abstract

Therapy-related myeloid neoplasms (t-MNs), which develop after cytotoxic, radiation, or immunosuppressive therapy for an unrelated disease, account for 7%–8% of acute myeloid leukemia (AML). Worse outcomes and consequently shortened survival are associated with t-MNs as compared with *de novo* AML. Therapy-related MNs are being reported with increasing frequency in successfully treated acute promyelocytic leukemia (APL), in particular, before the introduction of all-*trans* retinoic acid (ATRA) plus arsenic trioxide (ATO). Considering the high curability of APL, t-MNs represent one of the prognosis-limiting factors in this setting of leukemia. We report our experience with a patient who developed t-AML 15 years after treatment for APL. Treatment included three cycles of chemotherapy with CPX-351 (Vyxeos, Jazz Pharmaceuticals) followed, as in remission, by an allogeneic hematopoietic stem cell transplant. A review of available literature was also included.

## Introduction

Therapy-related myeloid neoplasms (t-MNs), including therapy-related myelodysplasia (t-MDS) and acute myeloid leukemia (t-AML), have been extensively reported after cytotoxic therapy or immunosuppressive treatment for solid tumors, lymphomas, or autoimmune disorders, more rarely after treatment for acute myeloid leukemia (1).

Traditionally subgrouped according to the previous exposure to alkylating agents, topoisomerase II inhibitors, or radiotherapy (RT), more recently, t-MN development has been associated with new agents belonging to different classes of chemotherapy (CHT) drugs, such as poly(ADP-ribose) polymerase inhibitors or purine analogs (2, 3).

Moreover, recent advances in deep sequencing techniques have significantly improved the knowledge of t-MNs over the last years, changing some of the classical views.

Acute promyelocytic leukemia (APL) is characterized by the translocations that fuse the *PML* gene on chromosome 15 to the *RARalpha* gene on chromosome 17 [t(15;17)], leading to a *PML*–*RARalpha* fusion gene; other peculiarities are the morphology of blast cells and a specific coagulopathy. Thanks to the advent of all-*trans* retinoic acid (ATRA) combined with anthracycline-based chemotherapy (4, 5) and/or arsenic trioxide (ATO), a cure rate higher than 70% has been achieved, even in relapsed patients (6–8). Thereby, the number of long-term survivors of this disease has increased over time. Consequently, more patients will be at risk of late complications related to antileukemic treatment.

Regarding t-MNs occurring after treatment for APL, sporadic cases have been reported in the literature, while only three major studies have assessed the incidence of t-MNs, ranging from 0.97% to 6.5% (4, 5). Moreover, only one of those studies calculated the cumulative incidence of a competing risk at a given time, resulting in approximately 2.2% at 6 years (9).

Survival in t-MNs is poor. In addition to the biology of t-MNs, the patient’s previous disease history and remission status at t-MN diagnosis are significant factors contributing to unfavorable outcomes. Also, t-MNs secondary to APL are usually difficult to treat, representing one of the prognosis-limiting factors for the curable APL disease.

We report a patient who developed t-AML 15 years after completion of maintenance therapy according to the GIMEMA AIDA2000 protocol for a previous APL still in molecular remission. A comprehensive review of the literature of previously published cases is also included.

## Case report

A 46-year-old man presented in January 2005 with fatigue, dyspnea, and a history of bleeding tendency. Coagulation tests showed disseminated intravascular coagulation, and peripheral blood cell count was as follows: hemoglobin 90 g/L, white cells 66.000 × 10^9^/L (with 60% hypergranular promyelocytes), and platelets 12.000 × 10^9^/L. Bone marrow revealed 70% hypergranular promyelocytes, with the characteristics t(15;17)(q22;q21) in all metaphases examined; molecular biology studies (performed by reverse transcription–polymerase chain reaction (RT-PCR)) confirmed the presence of *PML/RARα* gene rearrangement type bcr3. A diagnosis of high-risk hypergranular APL was made. Next-generation sequencing (NGS) analysis was not performed at diagnosis of APL.

The patient was treated according to the GIMEMA AIDA2000 protocol, receiving induction treatment with oral ATRA (45 mg/m^2^ per day for a total of 45 days) and four doses of intravenous idarubicin (12 mg/m^2^ on days 2, 4, 6, and 8): a complete molecular remission was achieved on day 38. Consolidation (according to a risk-adapted strategy) consisted of three courses, as follows: one course with intravenous cytosine arabinoside (Ara-C) (1 g/m^2^ on days 1, 2, 3, and 4) plus idarubicin (15 mg/m^2^ on days 1, 2, 3, and 4) plus oral ATRA (45 mg/m^2^ per day for 15 days); then, intravenous mitoxantrone (10 mg/m^2^ on days 1, 2, 3, 4, and 5) plus etoposide (100 mg/m^2^ on days 1, 2, 3, 4, and 5) plus oral ATRA (45 mg/m^2^ per day for 15 days); finally, intravenous idarubicin (12 mg/m^2^ on days 1) plus Ara-C (150 mg/m^2^ every 8 hours on days 1, 2, 3, 4, and 5) plus 6-thioguanine (70 mg/m^2^ every 8 hours on days 1, 2, 3, 4, and 5) plus oral ATRA (45 mg/m^2^ per day for 15 days).

Then, as in molecular remission, maintenance therapy was started, consisting of intramuscular methotrexate (15 mg/m^2^) plus oral 6-mercaptopurine (50 mg/m^2^) alternating with oral ATRA (45 mg/m^2^ per day for 15 days) every 3 months for a total of 2 years.

Annual cytogenetic and molecular analyses were performed until December 2015, confirming molecular remission. From January 2018 to February 2020, the patient stopped his annual follow-ups. In March 2020, blood cell count revealed mild anemia (hemoglobin 120 g/L) and thrombocytopenia (platelets 111.000 × 10^9^/L). Bone marrow analysis, performed in May 2020, confirmed molecular remission with initial cytological signs of dysplasia. Blood cell count remained stable until May 2022, when a morphological analysis of peripheral blood detected almost 10% blast cells. Bone marrow aspiration was hypercellular, showing 60% blast cells and red-cell line hyperplasia with multiple dyserythropoietic changes in erythroblasts (megaloblastic features, abnormal mitosis, and lobulated nuclei). Cytogenetic analysis revealed a complex karyotype (47, XY, +8, −2, −5, ins(mar;9)(?;q)?, del(12)(p13), +mar, inc), without t(15;17)(q22;q21). The molecular biology study was negative for *PML/RARα* gene rearrangement and positive for *WT1* gene hyperexpression and *KIT-D816V* exon 17 mutation.

NGS analysis, performed using second-generation sequencing technology on an Illumina MiSeq System (Illumina, San Diego, CA, USA) high-throughput sequencing platform, showed TP53 positivity with a variant allele frequency (VAF) of 78.0%.

Treatment with CPX-351 (Vyxeos, Jazz Pharmaceuticals, Dublin, Ireland; a liposomal encapsulation of cytarabine and daunorubicin in a synergistic 5:1 drug ratio) was started on June 2022—when the patient was 63 years old. CPX-351 has a specific indication for newly diagnosed s-AML, including t-AML, and the choice of CPX-351 was also linked to the age of the patient, good performance status, and time to previous treatment.

Before starting treatment, the patient had a normal echocardiogram [left ventricular ejection fraction (LVEF) 68%] and spirometry (diffusing capacity of the lungs for carbon monoxide (DLCO) 85%) and was considered fit for an intensive chemotherapy program. A total of three cycles of CPX-351 were administered (first and second induction and then consolidation), all well tolerated.

**Figure 1:**
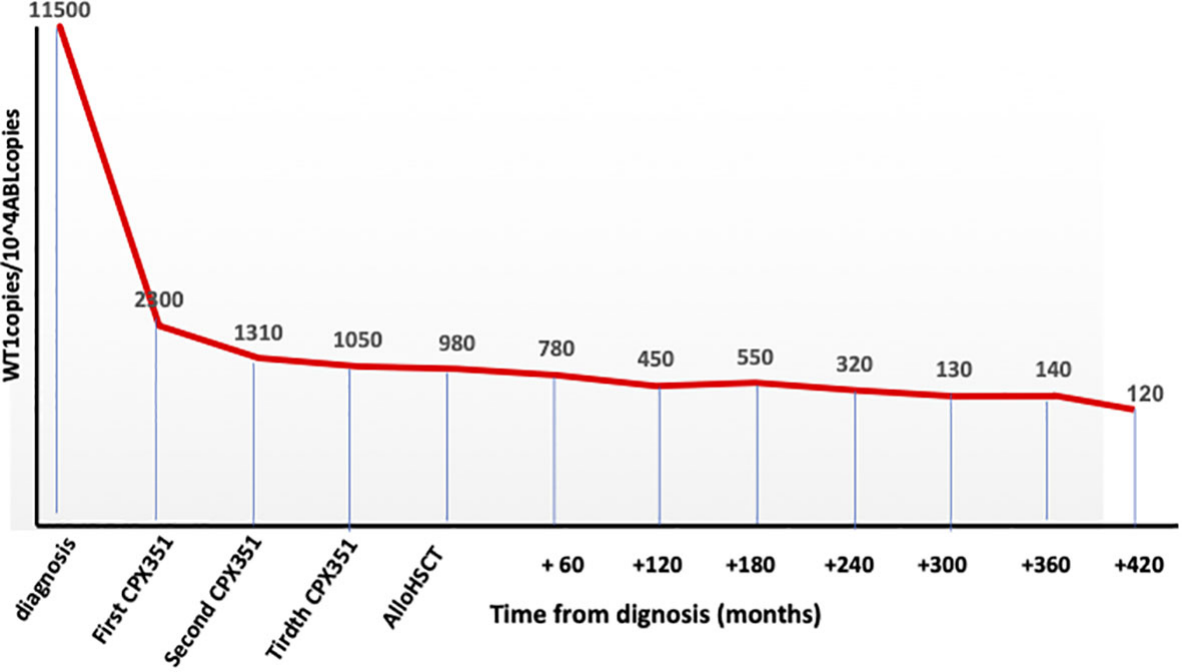
*WT1* clearance during the whole program.

Cytofluorimetric remission but not a complete clearance of *WT1* gene hyperexpression (**Figure 1**) was obtained after the first CPX-351 cycle and then maintained during the other two cycles.

In October 2022, as still in cytofluorimetric remission but with *WT1* over conventional threshold limits (**Figure 1**), an allogeneic hematopoietic stem cell transplant from an unrelated donor was performed. The patient was 64 years old at transplant with a hematopoietic cell transplant-specific comorbidity index (HCT-CI) score of 3 (previous leukemia) (10).

The conditioning regimen consisted of treosulfan i.v. plus fludarabine i.v.; graft versus host disease (GVHD) prophylaxis consisted of sirolimus, mycophenolate, and post-transplant cyclophosphamide. Allogeneic peripheral blood stem cells were infused on October 15, 2022.

The patient developed acute and then chronic skin GVHD, treated and resolved by steroid and extracorporeal photopheresis. Immunosuppressive treatment was completely withdrawn in March 2023. At the last follow-up—August 31, 2023—the patient was alive, with a mild chronic GVHD (mouth and skin), and in molecular remission with a full donor chimerism.

## Review of the literature

A total of 57 t-MN cases secondary to APL treatment were reported in the literature from 1992 to 2010: 44 (77.0%) patients were diagnosed with t-MDS and 13 (23.0%) with t-AML. After 2010, no other t-MN cases secondary to APL treatment were reported in the literature.

The main characteristics of the 57 patients are listed in **Table 1** (t-MDS) and **Table 2** (t-AML); the median age at diagnosis of APL was 51.5 years (8–73).

**Table 1:** Main characteristics and clinical course of patients developing t-MDS after APL therapy.

Ref. n.	Karyotype at APL diagnosis	Therapy for APL				Time to t-MDS (months)	PML-RARα at t-MDS (RT-PCR)	Karyotype at t-MDS diagnosis	Time to t-AML (months)	Therapy for t-AML
		ATRA	Anthracycline	Etoposide	Alkylating agent					
(11)	t (15;17) (q22;q21)		#	#		33	Negative	t (7;21) (q31;q22)	11	ATRA+CHT
(12)	t (15;17) (q22;q21)		#		#	24	Negative	45,XX,dic (5;17) (q11;p11/43,idem, −7, −20	2	NA
(13)	t (15;17) (q22;q21)	#	#			34	NA	45, XX, −7, der (7)del (7) (p10), del (7) (q21)	No t-AML	CHT
(14)	t (15;17) (q22;q21)	#	#	#		25	Negative	47, XY, +8	10	CHT+autoHSCT
(15)	t (15;17) (q22;q21)		#	#		84	NA	43, XX, del (5) (q15), −7, add (9) (q34), −18, −21	No t-AML	NA
(16)	t (15;17) (q22;q21)	#	#	#		26	Negative	−5, add (6)/ (p23-25), +8, add (17) (p23)	4	CHT
(17)	t (15;17) (q22;q21)	#	#			32	Negative	45, XX, −5, add (17) (p11.2), del3 (p23p25), −5, der (6), t (6;15) (p21;q15), −15, add17 (p13)	No t-AML	AlloHSCT upfront
(18)	t (15;17) (q22;q21)	#	#			35	Negative	45, XX, del (4) (q31), −5, add (5) (q35), −7, der (17)t (17);? (p11);?,-18, +mar1, +marX2[cp21]/46,XX (4)	No t-AML	CHT+alloHSCT
(4)	t (15;17) (q22;q21)	#	#			43	Negative	45, XX, −7	18	Supportive therapy
(4)	t (15;17) (q22;q21)	#	#			46	Negative	Failure	1	Supportive therapy
(5)	46, XX, del (3) (q24,q26), del (5) (q23q32), t (7;11) (p11;p12), t (15;17) (q22;q21)	#	#			32	Negative	45, XX, del (5) (q21q34), −7	7	CHT
(5)	46, XY, del (9) (q21q31), t (15;17) (q22;q210	#	#			111	Negative	45, XY, −5, der (7)t (7;20) (q11;p?orq)?,der (10)t (7;10;20) (q3?;q2?;p?orq)?,-13, der (17)t (10;17) (q2?;p11),-20,del (20) (q11),+mar1,+mar3/47,idem,del (X) (q26), der (1) (1);? (p36);?,+8,+mar2	No t-AML	Supportive therapy
(5)	failure	#	#			74	Negative	45, XY, −8, t (8;11) (q32;q21)	18	Supportive therapy
(5)	t (15;17) (q22;q21)	#	#			47	Negative	45, XY, t (3;17) (p11;q11),del (5) (q13q33), del (6) (p22),-17	No t-AML	CHT
(19)	t (15;17) (q22;q21), inv (6) (p24q13)	#	#			4	Negative	44, X, −Y, −7	6	Supportive therapy
(19)	t (15;17) (q22;q21)	#	#			20	Negative	46, XX, del (5) (q13,q33)	No t-AML	Supportive therapy
(20)	t (15;17) (q22;q21)	#	#			168	Negative	47, XY, +1, i (1) (q10) (21)/46,XY (4)	No t-AML	Supportive therapy
(22)	t (15;17) (q22;q21)	#	#			18	Negative	46, X, del (X) (q22q28),t (2;11) (q37;q23),del (7) (q22q36)	No t-AML	alloHSCT upfront
(9)	48, XY, t (15;17) (q22;q21), +21,+mar	#	#			52	Negative	45, XY, −7	19	Azacytidine than CHT
(9)	Failure	#	#			62	Negative	45, XX, del (5) (q13;q32), add (10) (p15),der (11) (q)?,add (12) (p13),add (12) (q)?,-13,-18,+mar	6	CHT+alloHSCT
(9)	Failure	#	#			23	Negative	Failure	No t-AML	CHT+alloHSCT
(9)	t (15;17) (q22;q21)	#	#			48	Negative	46, XX, del (7) (q23), del (5), iso (17q)	6	Supportive therapy
(9)	t (15;17) (q22;q21)	#	#			23	Negative	46, XX, del (7q) (q23), t (2;11) (q37;q23),del (X) (q22)	9	CHT+alloHSCT
(9)	t (15;17) (q22;q21)	#	#			44	Negative	44,XY,del (5) (q13q33),-7,-18,add (20) (q13.3), add (11) (p11.2)	4	CHT
(9)	t (15;17) (q22;q21)	#	#			33	Negative	44, YX, −5,add (12) (p13),add (7) (q32),-19	6	Supportive therapy
(9)	t (15;17) (q22;q21), add (7q)	#	#			45	Negative	45, XX, −7, t (12;18) (p12;q21)	18	CHT+alloHSCT
(9)	t (15;17) (q22;q21)	#	#			56	Negative	Failure	No t-AML	Supportive therapy
(9)	Failure	#	#			41	Negative	46, XX, −7	13	CHT+alloHSCT
(23)	t (15;17) (q22;q21)	#	#	#	#	38	Negative	45, XY, −7	NA	NA
(24)	47, XX, +8, t (15;17) (q22;q21)	#	#			29	NA	45, XX, −5, −7, +11	No t-AML	ATRA
(4)	t (15;17) (q22;q21)	#	#	#	#	48	Negative	del (5) (q)?	2	Supportive therapy
(4)	t (15;17) (q22;q21), add (7q)	#	#	#	#	24	Negative	46, XX	5	AlloHSCT upfront
(5)	t (15;17) (q22;q21)	#	#			13	Negative	46, XX, del (5) (q22q34), t (15;21) (p11;q21), −17, +mar	No t-AML	Supportive therapy
(5)	t (15;17) (q22;q21)	#	#		#	46	NA	46, XY, del (5) (q12q35), add (11) (q23), dup (12) (q12q22), −17, −18, −22	1	Supportive therapy
(25)	46, XX	#	#	#		NA	Negative	45, XX, −7/46,idem,+21 RUNX1D171N; NRASG12V	t-AML	CHT
(25)	t (15;17) (q22;q21)	#	#	#		NA	Negative	45, XY, −7/46, XY RUNX1D171G	t-AML	CHT
(25)	t (15;17) (q22;q21)	#	#	#		NA	Negative	46,XY, t (7;15) (q11;q11), der (12)t (12;17) (p11;q21), t (16;21) (q24;q22), add (17) (q11), add (19) (p13), del (21) (q21), 46,idem,der (18)t (15,18) (q11;p11)/46,XY RUNX1MTG16	t-AML	CHT
(25)	t (15;17) (q22;q21)	#	#	#		NA	Negative	46, XX, t (6;11) (q21;q23) MLL-FOXO3	t-AML	CHT
(25)	t (15;17) (q22;q21)	#	#	#		NA	Negative	46, XY, aad (2) (p23), inv (5) (p11q23),add (11) (q23)/46,idem,inv (2) (p23q11)/47,idem,+13 RUNX1S295fsX571	t-AML	CHT
(25)	t (15;17) (q22;q21)	#	#	#		NA	Negative	45, XY,-7, RUNX1G172W	t-AML	CHT
(25)	t (15;17) (q22;q21)	#	#	#		NA	Negative	46,XY,t (11;16) (q23;p13.3) MLL-CBP; FLT3ITD	t-AML	CHT+alloHSCT
(25)	t (15;17) (q22;q21),	#	#	#		NA	Negative	46, XY CEBPAQ305P	t-AML	CHT
(25)	t (15;17) (q22;q21)	#	#	#		NA	Negative	46, XX, del (20) (q11)	No t-AML	Supportive therapy
(25)	t (15;17) (q22;q21)	#	#	#		NA	Negative	46, XX, del (20) (q1)?	No t-AML	Supportive therapy

APL, acute promyelocytic leukemia; ATRA, all-trans retinoic acid; autoHSCT, autologous hematopoietic stem cell transplant; alloHSCT, allogeneic hematopoietic stem cell transplant; CHT, chemotherapy; NA, not available; t-AML, therapy-related acute myeloid leukemia; t-MDS, therapy-related myelodysplastic syndrome.

#=yes; ?= symbols of cytogenetic.

**Table 2:** Main characteristics and clinical course of patients developing t-AML after APL therapy.

Ref. n.	Karyotype at APL diagnosis	Therapy for APL				Time to t-AML (months)	PML-RARα at t-MDS (RT-PCR)	Karyotype at t-AML diagnosis	Therapy for t-AML
		ATRA	Anthracycline	Etoposide	Alkylating agent				
(26)	NA		#	#		43	NA	t(3;21)(q26;q22), der(4)t(4);?(q27);?,der(7)t(4;7)(q27;q22), der(16)t(16);?(p11);?	ATRA
(27)	t(15;17)(q22;q21)		#	#	#	43	NA	t(10;11)(q23;p15)	CHT
(28)	t(15;17)(q22;q21)	#	#	#	#	34	NA	45, XY, −7	CHT
(29)	t(15;17)(q22;q21)	#	#	#		49	Negative	t(10;11)(p14;q21)	CHT+alloHSCT
(30)	Failure	#	#			12	Negative	46,XX,t(8;16)(p11.2;p13.3), inv(11)(p15q22-q23) (31);47,idem,+i(8)(q10) (9)	CHT
(9)	Failure	#	#			39	Negative	46, XX	Supportive therapy
(9)	t(15;17)(q22;q21)	#	#			43	Negative	45, XX, −5, add(17)	CHT
(9)	t(15;17)(q22;q21)	#	#			54	Negative	55, X, der(Y), t(Y;10)(p11;q11),add(1p),+4,+9,+11,+17,-18,+20,+21,add(22q),+3mar	CHT+alloHSCT
(9)	t(15;17)(q22;q21)	#	#			24	Negative	46, XY, t(9;11)(p22;q23)	CHT+alloHSCT
(9)	Failure	#	#			42	Negative	failure	CHT+alloHSCT
(9)	t(15;17)(q22;q21)	#	#			52	Negative	46, X, −Y, +8	CHT+alloHSCT
(9)	Failure	#	#			17	Negative	Failure	CHT
(25)	t(15;17)(q22;q21)	#	#	#		80.4	Negative	46, XY, add(13)(q32) -	CHT

APL, acute promyelocytic leukemia; ATRA, all-trans retinoic acid; autoHSCT, autologous hematopoietic stem cell transplant; alloHSCT, allogeneic hematopoietic stem cell transplant; CHT, chemotherapy; NA, not available; t-AML, therapy-related acute myeloid leukemia; t-MDS, therapy-related myelodysplastic syndrome.

#=yes; ?= symbols of cytogenetic.

**Table 3** summarizes the clinical and treatment characteristics of the whole population.

**Table 3:** Main characteristics: treatment and outcome of the whole population.

**N. patients**	
• t-MDS • t-AML	44 (77.0%) 12 (23.0%)
Sex	
• Male • Female	28 (49.0%) 29 (51.0%)
**Median age, years (range) at diagnosis of APL** **Median age, years (range) at diagnosis of t-MNs**	51.5 (8.0–73.0) 55.2 (26.0–78.0)
**Median time to t-MDS, months (range)—44 patients** **Median time from t-MDS to t-AML, months (range)—28 patients** **Median time to t-AML, months (range)—13 patents**	39.5 (4.0–168.0) 6.5 (1.0–19.0) 43.0 (17.0–54.0)
**Cytogenetic at diagnosis of t-MNs**	52 (91.0%) patients
Normal −5/del(5q) −7/del(7) Complex 21q22 11q23	3 (5.5%) 18 (31.5%) 21 (37.0%) 24 (42.0%) 9 (15.5%) 8 (14.0%)
Treatment for APL	
Anthracycline Etoposide 6-Mercaptopurine plus mitoxantrone 6-thioguanine Alkylating agent (autoHSCT)	57 (100%) 22 (38.5%) 42 (73.5%) 8 (14.0%) 7 (12.0%)
Treatment for t-MNs	
Supportive therapy ATRA alone Conventional CHT AlloHSCT NA	16 (29.5%) 2 (3.5%) 33 (61.0%) 15 (29.5%) 3 (5.0%)

APL, acute promyelocytic leukemia; ATRA, all-trans retinoic acid; autoHSCT, autologous hematopoietic stem cell transplant; alloHSCT, allogeneic hematopoietic stem cell transplant; CHT, chemotherapy; NA, not available; t-AML, therapy-related acute myeloid leukemia; t-MDS, therapy-related myelodysplastic syndrome.

In all 57 patients, RT-PCR monitoring and/or cytogenetic analysis indicated molecular remission of APL at diagnosis of t-MNs. Overall, the median time from the achievement of remission to diagnosis of t-MN was 42.5 months (4–168).

No significant statistical difference between t-MDS and t-AML was observed in the time from the first complete response (CR) to the development of t-MNs (t-MDS: 39.5 months (4–168) *vs.* t-AML: 43 months (17–54), p = 0.07).

Using conventional karyotyping or fluorescent *in situ* hybridization, cytogenetic characterization was successful in 52 (91.0%) of 57 patients and was abnormal in all except three cases, with complex karyotypes (≥three independent abnormalities) observed in 24 (42.0%) patients (**Table 3**).

### Treatment and clinical course of t-MN

Except for three patients for whom treatment was not included in the report, in all the other 54 patients, therapy for t-MN consisted of only supportive therapy in 16 patients (29.5%) (15 MDS and one AML); all of them died after a median of 9 months from t-MN diagnosis (range, 1 to 39 months). The majority of patients (33%–61.0%) were treated with conventional chemotherapy (in one patient, an autologous stem cell transplant was performed after induction treatment): for two patients, follow-up was not available, and all the others died of progressive disease.

Allogeneic stem cell transplant (alloHSCT) was performed in 15 (29.5%) patients (three patients up-front and 11 patients after induction chemotherapy): four transplanted patients lived more than 12 months from transplant, but follow-up was not subsequently updated, while all the others died due to transplant-related mortality (five patients) or progressive disease (six patients) within 12 months from reinfusion (**Table 3**).

## Discussion

Therapy-related AML after APL treatment is a relatively infrequent (<7.0%) and late complication bearing a poor prognosis (4, 5).

Incidences reported in the largest studies ranged between 0.97% (European APL study: a series of 617 patients with a median follow-up of 51 months) and 6.5% (Italian study of 46 patients: follow-up not reported) (4, 5). Because the risk of developing t-AML continues for many years after the end of treatment, the PETHEMA group evaluated the cumulative incidence of t-AML in patients enrolled in three consecutive trials (LPA96, LPA99, and LPA2005): 918 patients were observed for a median of 77 months with a cumulative incidence of t-AML of 2.2% at 6 years, not comparable with the crude incidence of the other two studies (9).

The introduction of ATO in combination with ATRA had further reduced the incidence of t-AML in the APL setting, as reported by the Italian-German APL0406 study, where, with a follow-up of 6 years; no t-MN cases were observed in ATRA/ATO group patients *vs.* 1.5% in those treated with the AIDA regimen (10). Similar results have been reported from the AML17 trial (National Cancer Research Institute): with a follow-up of 5.7 years, no t-AMLs were observed in the ATRA/ATO group *vs.* 1.0% in the AIDA group (32).

Cytogenetic abnormalities have been largely described in patients with t-AML: a decreased prevalence of normal karyotype (<30.0%) and a prevalence of complex or unbalanced karyotypes with chromosomal deletions as compared with the *de novo* AML were reported (2, 21, 33–35). The combination of multiple chemo-immunotherapy agents with different mechanisms of action makes it difficult to ascribe the mutagenic potential to a single drug. Traditionally, recurrent translocations as t(15;17), t(8;21), inv(16), t(15;17), and 11q23 abnormalities were associated with topoisomerase II inhibitors, and t-AML usually developed after a latency time of 1 to 3 years (36–38). Very complex karyotypes (>5 simultaneous chromosomal abnormalities) and deletions of chromosomes 5 and 7 were usually associated with alkylating agents or radiotherapy, occurring after a latency of 5 to 7 years (2, 39, 40).

Therapy-related MDS is usually characterized by very complex karyotypes and consequently by a poor and very poor cytogenetic risk (40). Therefore, according to the Revised International Prognostic Scoring System (IPSS-R), a high prevalence of high- and very-high-risk subgroups was expected in the t-MDS setting. The IPSS-R is applicable to t-MDS and *de novo* MDS and reliably predicts AML transformation. As reported in the literature, 28 of the 44 (63.5%) t-MDS cases that developed after APL treatment subsequently progressed to t-AML at a median time of 6.5 months (1–19) (**Tables 1**, **3**).

The molecular characteristics of MNs have been extensively analyzed in recent years: in more than 95% of AML and MDS, somatic mutations have been detected, without significant difference in the overall number of mutations in secondary *vs. de novo* subtypes. Moreover, none of the genes were exclusively mutated in t-AML. Mutations in RNA-splicing genes, epigenetic regulators genes, or cohesin complex genes were more than 90.0% specific for the diagnosis of s-AML (39, 41) and were present in only 30.0% of t-AML.

In the 57 t-MN cases that developed after APL treatment reported in the literature, in addition to an anthracycline (all the 57 patients), 22 (38.5%) patients also received etoposide, 42 (73.5%) received 6-mercaptopurine plus mitoxantrone as maintenance treatment, and eight (14.0%) received 6-thioguanine. Only seven patients (12.0%) received an alkylating agent as a part of the conditioning regimen for autologous stem cell transplantation.

Concerning cytogenetic analysis, in the 57 cases reported in the literature (**Table 3**), balanced translocations that involved 21q22 and 11q23 (typical breakpoints observed in t-AML occurring after administration of topoisomerase II inhibitors) were detected in nine (15.5%) and eight (14.0%) patients, respectively (**Table 3**). Moreover, 18 (31.5%) patients had −5/del(5q), and 21 (37.0%) had −7/del(7) abnormalities. Complex karyotypes (≥three independent abnormalities) were revealed in 24 (42.0%) patients (**Table 3**).

No NGS analysis was performed in the 57 t-MN cases reported in the literature, while in 11 patients (19.5%), molecular analysis by RT-PCR was reported (ref (25), **Tables 1**, **2**).

Our patient was extensively studied by RT-PCR and NGS at diagnosis of t-AML, confirming the absence of *PML/RARα* gene rearrangement and presence of *KIT-D816V* exon −17 mutation and TP53 gene mutation, with a VAF of 78.0%.

In an independent series, mutations of *TP53* were reported in 30.0% to 47.0% of cases of t-MNs, resulting in the single most frequent molecular abnormality in this setting associated with complex karyotype in almost 80.0% of cases (39, 42–44). Lindsley et al. showed that TP53 mutations define a specific subgroup of t-AML, which differs from other AMLs like s-AML, in terms of younger age, lower recurrent driver mutations, more cytogenetic abnormalities, and poor prognosis with a reduced probability of achieving response after conventional treatment (39).

KIT mutations are detected in approximately 4%–6% of adult patients with *de novo* AML (45, 46) and 20%–40% of adult patients with *de novo* core-binding factor (*CBF*) leukemia (47–51). Three mutational hot spots (exon 8, exon 10–11, and exon 17) have been identified in the *KIT* gene (37, 52–54). Of these, exon 17 (detected in our patient) represents the site of *KIT* mutations most strongly associated with poor prognosis.

As KIT mutations have been reported mostly in *CBF*-AML, most studies on *KIT* mutations have been limited to *CBF*-AML, with few studies investigating *KIT* mutations in t-MNs. Schnittger et al. performed a large-scale study involving almost 2,000 unselected patients with AML: among 125 t-AML patients of the series, *KIT* mutation was detected in only one patient, who also presented t(8;21) translocation (54). Another study on 140 patients with t-MNs reported two cases with *KIT-D816V* mutation, one of which had t(8;21) (55).

*KIT* and *TP53* mutations were not detected together in any of the cases reported in the literature. Survival in t-MNs is poor when compared with that in other leukemia subtypes: until recent years, patients with t-MNs have been conventionally excluded from many clinical trials. This is particularly relevant in patients with previous APL, which is now considered a curable disease in many patients. New drugs with specific activity on secondary leukemia (including t-AML), targeting pathogenic mutations or interfering with immune mechanisms, are or will be available in the future. Our patient was treated with CPX-351 (Vyxeos, Jazz Pharmaceuticals): up to now, no other cases treated with CPX-351 and allogeneic stem cell transplant for a t-AML that developed after treatment according to GIMEMA AIDA2000 protocol have been reported in the literature.

The risk of anthracycline-induced heart failure increases as the cumulative dose administered increases: 3%–5% at 400 mg/m^2^ and as high as 18%–48% at 700 mg/m^2^ (56). However, there is a different level of risk for each patient scheduled for anthracycline therapy: patients younger than 5 years or older than 65 years, with prior or concurrent chest irradiation, pre-existing heart disease, or already known cardiovascular risk factors, have an increased risk of cardiotoxicity.

Our patient was 46 years old when he was treated according to GIMEMA AIDA2000 protocol: the anthracycline cumulative dose administered (as by protocol) was 600 mg/m^2^, and no concomitant cardiovascular risk factors were present at diagnosis, but unfortunately, LVEF before treatment was not available.

Before starting treatment for t-AML, our patient was 63 years old, without cardiac dysfunction (LVEF 68%), hypertension, or other cardiovascular risk factors.

As mentioned, CPX-351 is a liposomal encapsulation of cytarabine and daunorubicin: in the heart, liposomes cannot get out of the vascular space because capillaries have tight junctions. As the tendency to accumulate in the heart cells is limited, this may reduce the risk of cardiotoxicity. On the contrary, the liposomes reach high concentrations in the tumor site, leaving the circulatory system where tumor growth damages the capillaries (56).

In our patient, no cardiac dysfunction or other cardiovascular diseases were developed during the treatment for t-AML (from induction to transplant).

Of the 57 t-MN patients reported in the literature (**Table 3**), 15 underwent allogeneic stem cell transplant; no details about disease status at transplant and at last follow-up were reported, particularly about the molecular response. In our patient, with a high-risk genetic profile (*TP53* and *KIT*-*D816V* exon −17 mutation), a molecular response was achieved with a transplant procedure and confirmed at the last follow-up. Of course, a longer follow-up would be needed for overall response and chronic GVHD assessment.

Considering the high curability of APL with excellent complete remission and long-term survival rates, it is necessary to try to reduce the incidence of t-MNs with a risk-adapted strategy and use chemotherapy-free regimens like ATO/ATRA.

## Data availability statement

The original contributions presented in the study are included in the article/supplementary material. Further inquiries can be directed to the corresponding author.

## Ethics statement

Written informed consent was obtained from the individual(s) for the publication of any potentially identifiable images or data included in this article.

## Author contributions

AS: Conceptualization, Writing – original draft, Writing – review & editing. MS: Investigation, Writing – review & editing. JP: Investigation, Writing – review & editing. RM: Investigation, Writing – review & editing. SP: Data curation, Writing – review & editing. GC: Data curation, Writing – review & editing. EP: Data curation, Writing – review & editing. PF: Data curation, Writing – review & editing. FC: Writing – review & editing. MG: Writing – review & editing. MB: Data curation.
